# Supplementary material for: Development and Evaluation of Different Electrospun Cysteamine-Loaded Nanofibrous Webs: A Promising Option for Treating a Rare Lysosomal Storage Disorder
Source: Pharmaceutics. 2024 Aug 9;16(8):1052. doi: 10.3390/pharmaceutics16081052 (PMC11360779; doi:10.3390/pharmaceutics16081052)
Supplement: Supplementary file 1 [file pharmaceutics-16-01052-s001.zip › pharmaceutics-3101302-supplementary.pdf]

Development and Evaluation of Different Electrospun Cysteamine-Loaded Nanofibrous Webs: A Promising Option for Treating a Rare Lysosomal Storage Disorder

Safaa Omer, Nándor Nagy, Balázs Pinke, László Mészáros, Adrienn Kazsoki, and Romána Zelkó

Supplementary data

Table S1. Composition and respective amount of polyvinyl alcohol (PVA) solutions

Polymer Composition	PVA grades	Polymer Concentration (% (w/w))	Formulation Code
PVA	Mw ~67 kDa	15	P1
		17.5	P2
		20	P3
	Mw ~130 kDa	13	P4
		14	P5
		15	P6
	Mw ~205 kDa	5	P7
		7.5	P8
		10	P9

Table S2. Composition and respective ratios of polyvinyl alcohol (PVA)/poloxamer 407 (PO-407) solutions

**Polymer composition**	**PVA Grades**	**Total polymer Concentration (% (w/w))**	**PVA: PO-407 (mass ratio)**	**Formulation Code**
PVA/PO-407	Mw ~67 kDa	17.5	85:15	PP1
			80:20	PP2
			75:25	PP3
	Mw ~130 kDa	14	85:15	PP4
			80:20	PP5
			75:25	PP6
	Mw ~205 kDa	7.5	85:15	PP7
			80:20	PP8
			75:25	PP9

Table S3. Composition and respective amount of polyvinyl alcohol (PVA)/poloxamer 407 (PO-407)/polysorbate 80 (PS-80) solutions

**PVA: PO-407 (mass ratio)**	**PVA Grades**	**Total polymer concentration (% (w/w))**	**PS-80 (% (w/w))**	**Formulation Code**
80:20	Mw ~67 kDa	17.5	1%	PPP1
			0.5%	PPP2
	Mw ~130 kDa	14	1%	PPP3
			0.5%	PPP4
	Mw ~205 kDa	10	1%	PPP5
			0.5%	PPP6

Table S4. composition of the different Tetraethoxysilane (TEOS)/polyvinyl alcohol (PVA) samples

**Sample code**		**TEOS: PVA (mass ratio)**			
		1:4	2:3	3:2	4:1
PVA (%(w/w))	10	S1	S2	S3	S4
	12	S5	S6	S7	S8
	14	S9	S10	S11	S12

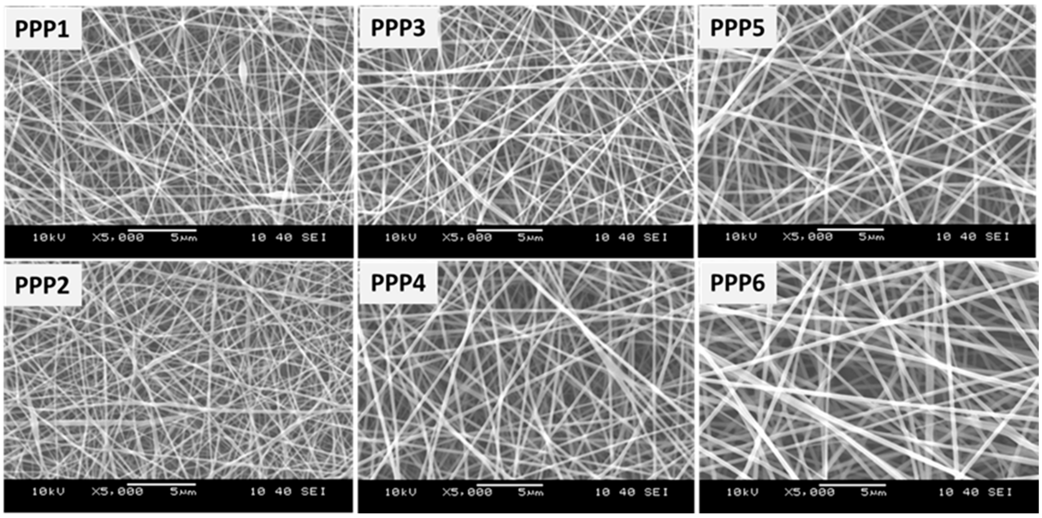

Figure S1. Scanning Electron Microscope (SEM) images of electrospun samples of polyvinyl alcohol (PVA)/poloxamer 407 (PO-407) blend with polysorbate 80 (PS-80) (0.5-1% (w/w)), (magnification: 5000×)

Table S5. Average fiber diameters of polyvinyl alcohol (PVA)/poloxamer 407 (PO-407)/polysorbate 80 (PS-80) nanofibers with respective skewness and kurtosis

Formulation code	PVA: PO-407 (mass ratio)	PVA Grades	Total polymer Concentration (%(w/w))	PS-80 (%(w/w))	Average fiber diameter (nm) ± SD (nm)	Skewness	Kurtosis
**PPP1**	80:20	Mw~67 kDa	17.5	1%	85 ± 20	0.21095	-0.23699
**PPP2**	80:20			0.5%	109 ± 13	0.113247	-0.86708
**PPP3**	80:20	Mw~130 kDa	14	1%	105 ± 21	0.230729	-0.01485
**PPP4**	80:20			0.5%	131 ± 23	-0.06011	-0.25659
**PPP5**	80:20	Mw~205 kDa	7.5	1%	127 ± 13	-0.02749	-1.16733
**PPP6**	80:20			0.5%	141 ± 14	-0.29537	-0.66186

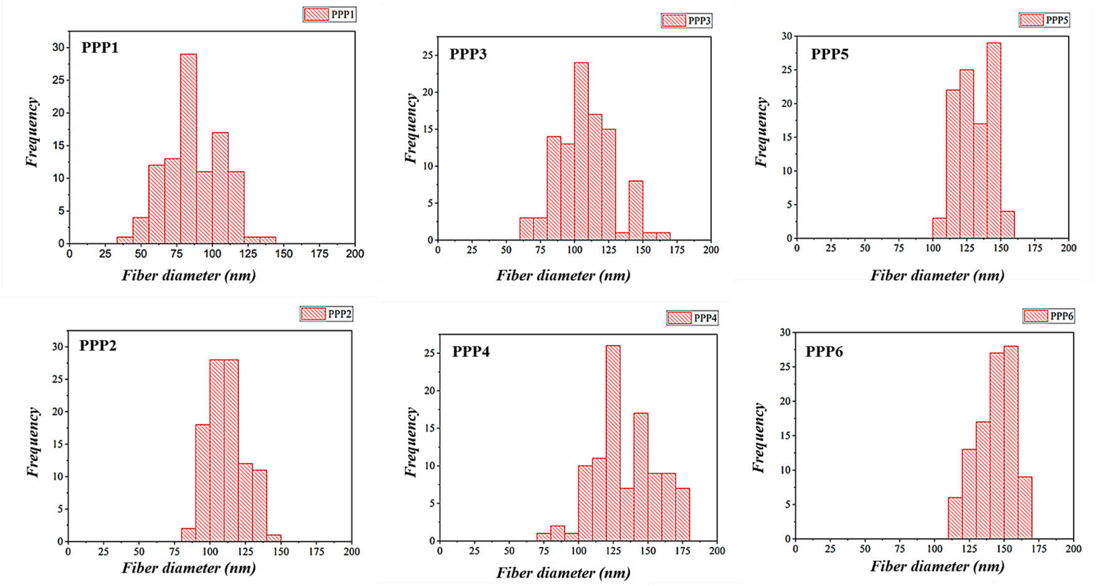

Figure S2. Fiber diameter distributions of electrospun polyvinyl alcohol (PVA)/poloxamer 407 (PO-407)/polysorbate 80 (PS-80) samples

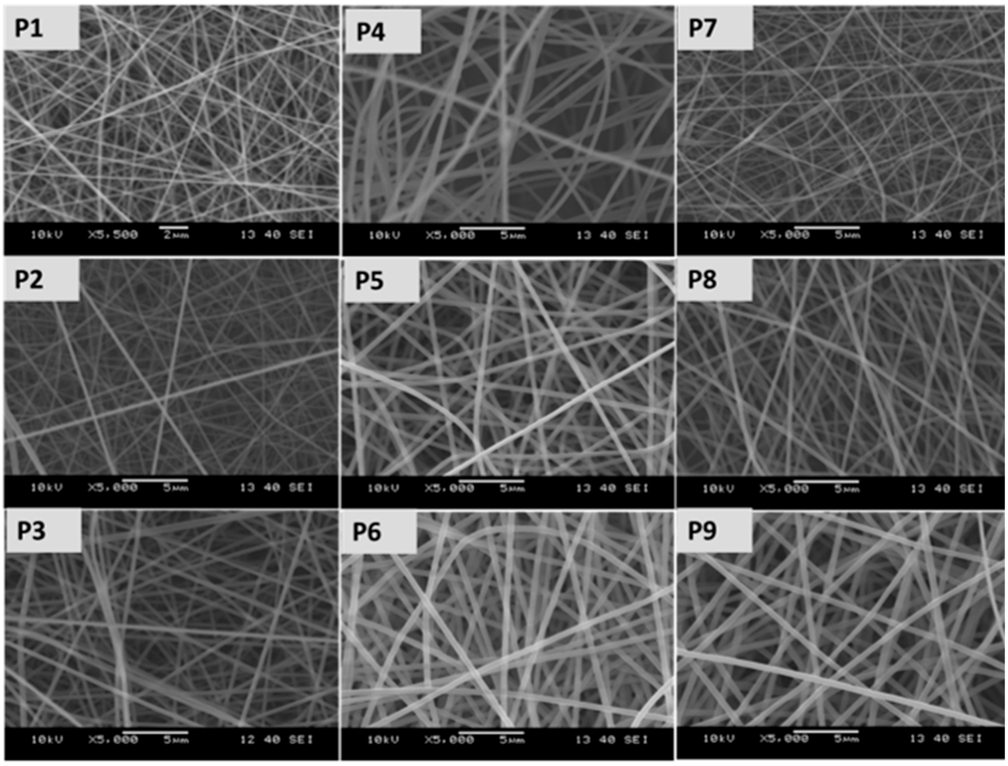

Figure S3. Scanning Electron Microscope (SEM) images of electrospun polyvinyl alcohol (PVA) samples prepared from different grades and concentrations of PVA (magnification: 5000×)

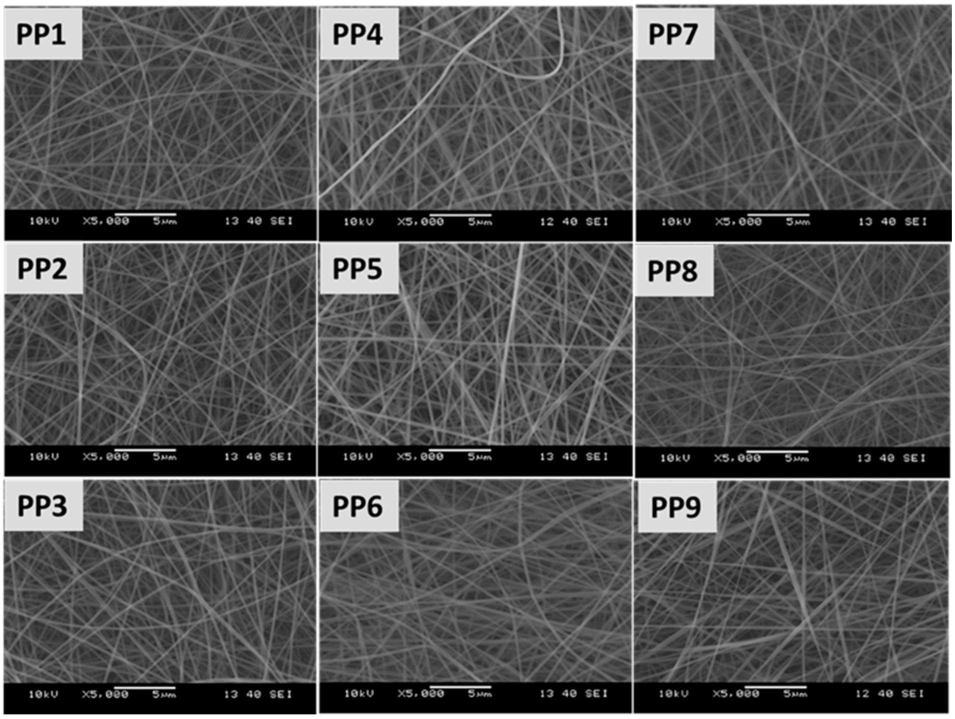

Figure S4. Scanning Electron Microscope (SEM) images of electrospun samples of different ratios of polyvinyl alcohol (PVA)/poloxamer 407 (PO-407) mixture (magnification: 5000×)

Table S6. Average fiber diameters of polyvinyl alcohol (PVA) nanofibers with respective skewness and kurtosis

PVA grades	Formulation Code	Polymer concentration (% (w/w))	Average fiber diameter (nm) ± SD (nm)	Skewness	Kurtosis
Mw ~67 kDa	P1	15	183 ± 27	0.648329	0.00709
	P2	17.5	190 ± 25	0.12191	0.055786
	P3	20	262 ± 34	-0.44344	-0.16322
Mw ~130 kDa	P4	13	283 ± 39	0.396731	-0.35636
	P5	14	324 ± 36	0.29524	-0.23358
	P6	15	389 ± 47	0.093752	-0.52959
Mw ~205 kDa	P7	5	170 ± 20	0.472034	-0.05075
	P8	7.5	296 ± 37	0.153405	-0.15343
	P9	10	425 ± 46	0.090873	-0.16408

Table S7. *Average fiber diameters of polyvinyl alcohol (PVA)/poloxamer 407 (PO-407) nanofibers with respective skewness and kurtosis*

Formulation Code	PVA Grades	Total polymer Concentration (%w/w)	PVA: PO-407 (m:m)	Average fiber diameter (nm) ± SD (nm)	Skewness	Kurtosis
PP1	M_w ~67 kDa	17.5	85:15	182 ± 28	1.204267	2.429205
PP2			80:20	173 ± 25	0.352606	-0.04599
PP3			75:25	167 ±19	0.847253	1.54499
PP4	Mw~130 kDa	14	85:15	195 ± 22	-0.28943	-0.78801
PP5			80:20	169 ±17	-0.17367	-0.9992
PP6			75:25	160 ± 16	0.325795	-1.2746
PP7	Mw~205 kDa	7.5	85:15	165 ±21	0.613269	1.345025
PP8			80:20	158 ± 15	0.216803	-0.87967
PP9			75:25	139 ± 29	0.418524	-0.48121

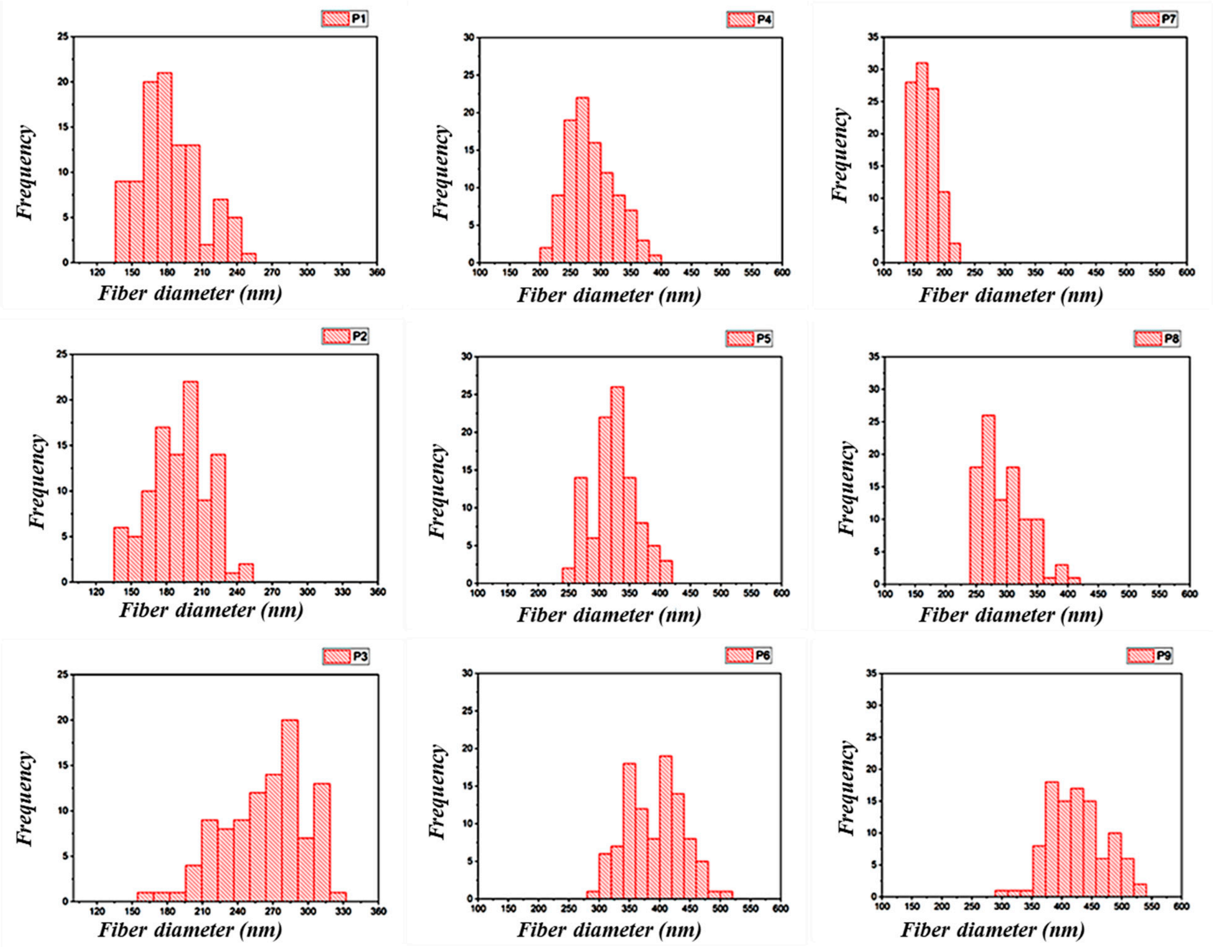

Figure S5. Fiber diameter distributions of electrospun polyvinyl alcohol (PVA) in different grades and concentrations

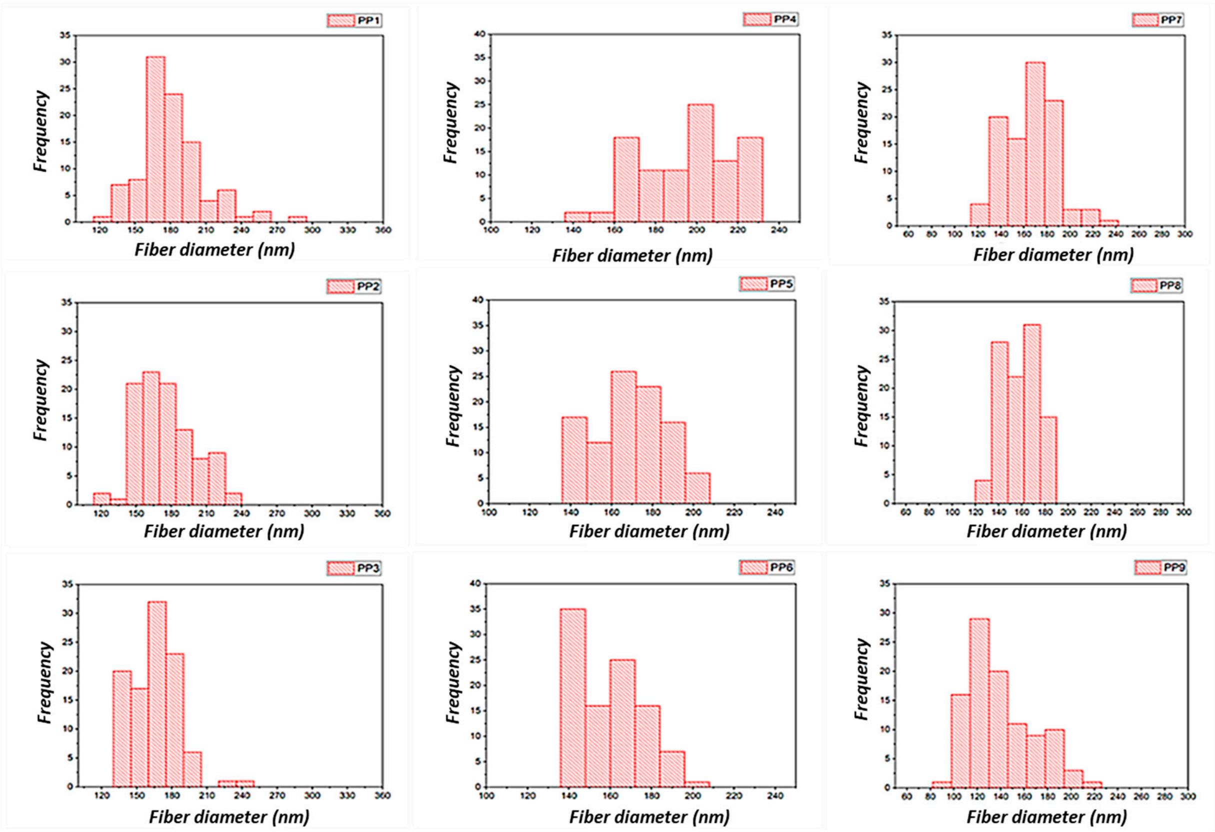

Figure S6. Fiber diameter distributions of electrospun polyvinyl alcohol (PVA)/poloxamer 407 (PO-407) samples of different PVA: PO-407 mass ratios

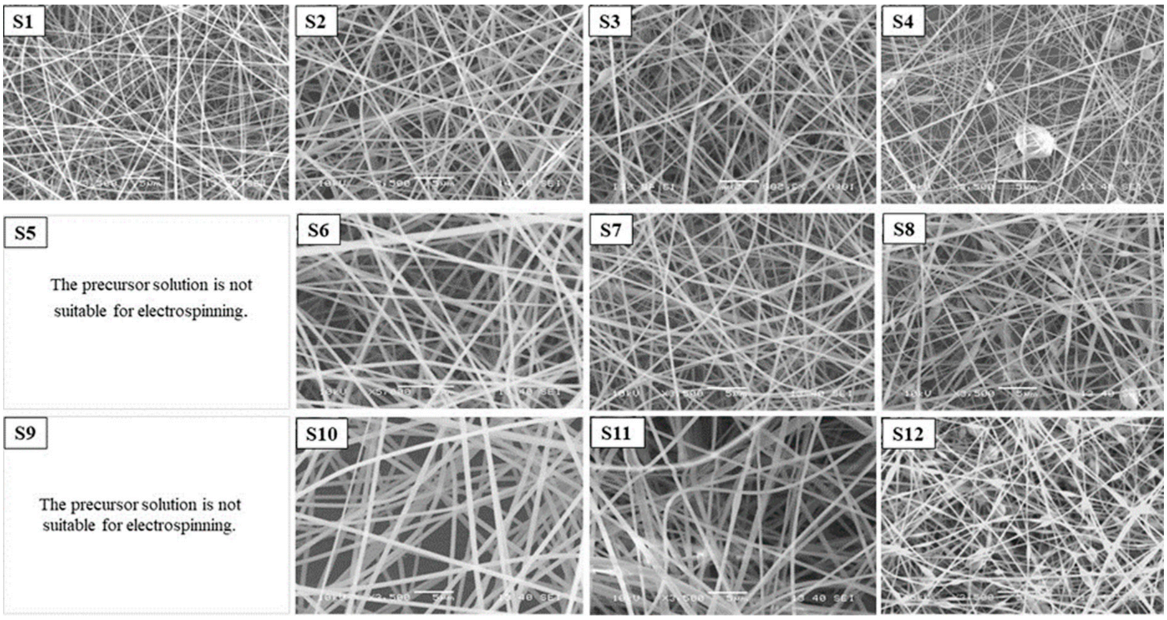

Figure S7. Scanning Electron Microscope (SEM) images of electrospun samples prepared from different compositions of tetraethoxysilane (TEOS)/polyvinyl alcohol (PVA) precursor solutions by electrospinning (Magnification: 3500x)

Table S8. The average fiber diameter values of the tetraethoxysilane (TEOS)/polyvinyl alcohol (PVA) samples

**Fiber diameter average (nm) ± SD (nm)**		**TEOS: PVA (mass ratio)**			
		**1:4**	**2:3**	**3:2**	**4:1**
C_{PVA} **(w/w%)**	**10%**	183 ± 33	282 ± 57	235 ± 51	129 ± 30
	**12%**	-	386 ± 48	298 ± 52	217 ± 58
	**14%**	-	572 ± 79	549 ± 83	244 ± 63

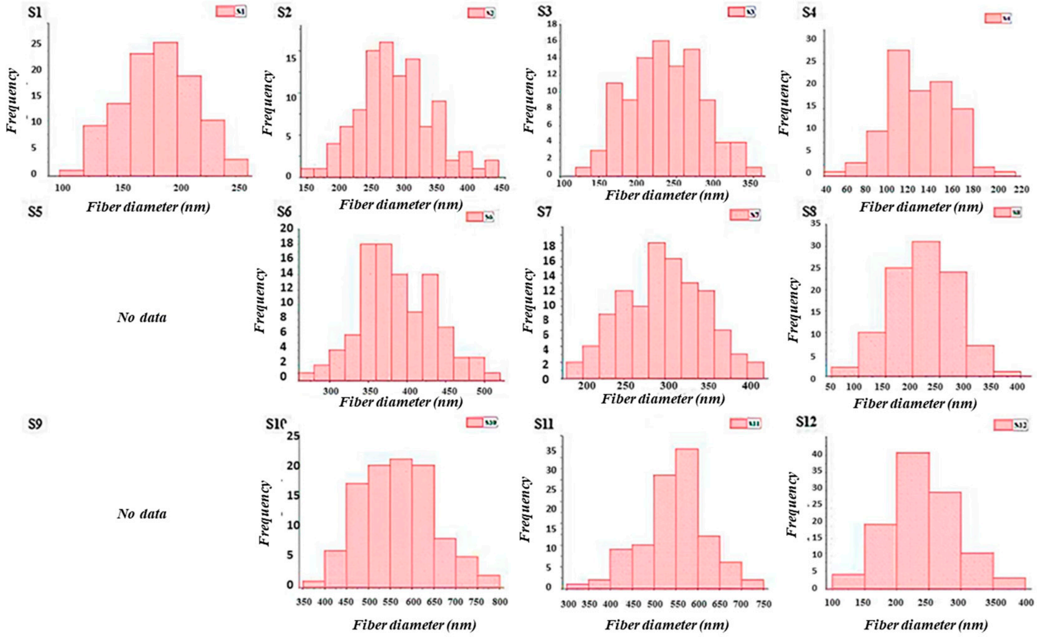

Figure S8. Fiber diameter distribution of the electrospun samples prepare from different tetraethoxysilane (TEOS)/polyvinyl alcohol (PVA)

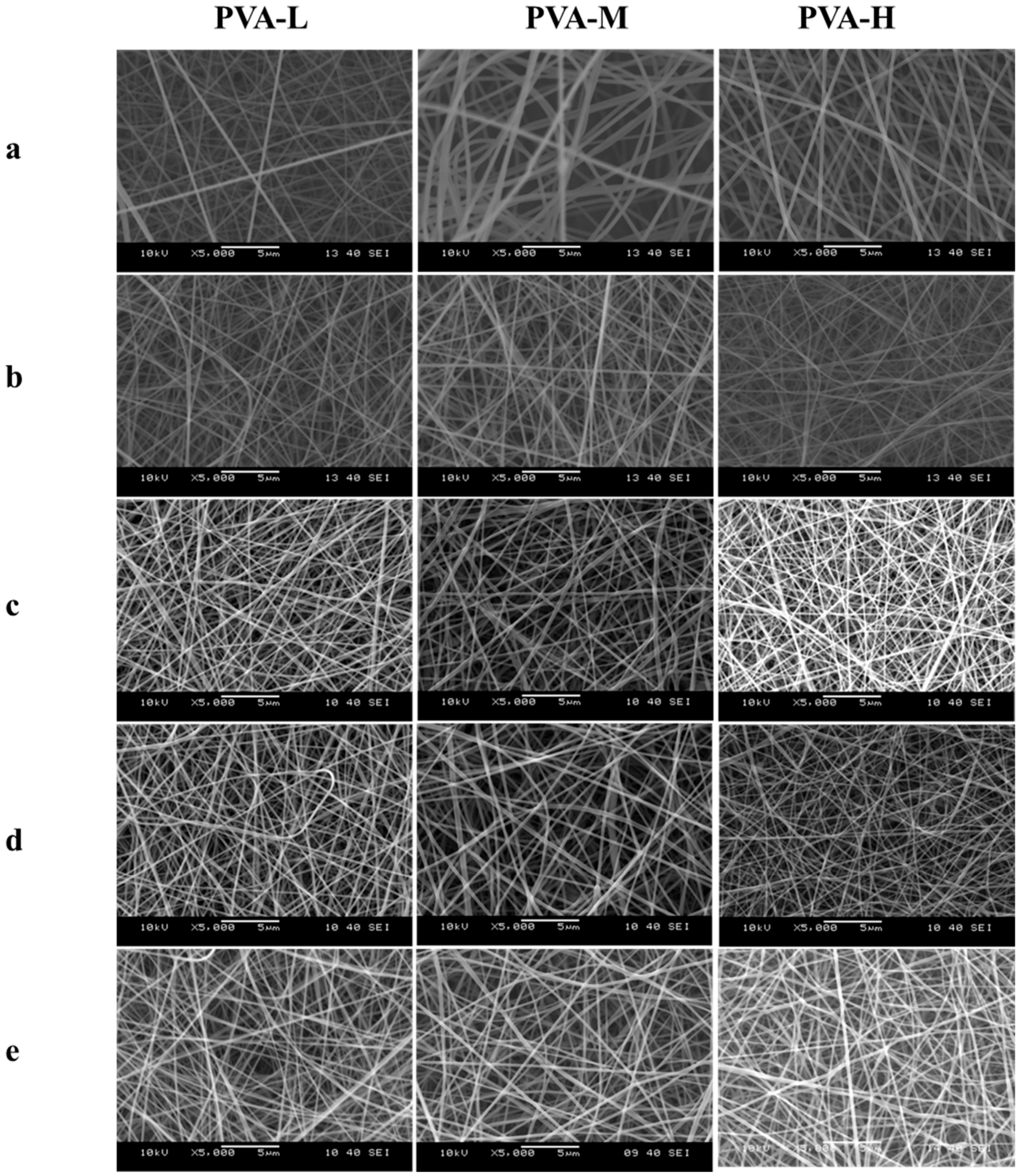

Figure S9. Scanning Electron Microscope (SEM) images of electrospun nanofibers obtained at 10 kV and 5000x magnification.

Formulations (PVA-L, PVA-M, and PVA-H; Polyvinyl alcohol (PVA) of Mw ~67 kDa, Mw ~130 kDa, and Mw ~205 kDa, respectively). a: plain PVA; b: PVA and poloxamer 407 (PO-407) mixture 80:20, c: 0.55%w/w cysteamine hydrochloride (CysH) in PVA/PO-407 matrix, d: component of c plus EDTA 0.01%w/w, e: component of d plus polysorbate 80 (PS-80) 1%(w/w))

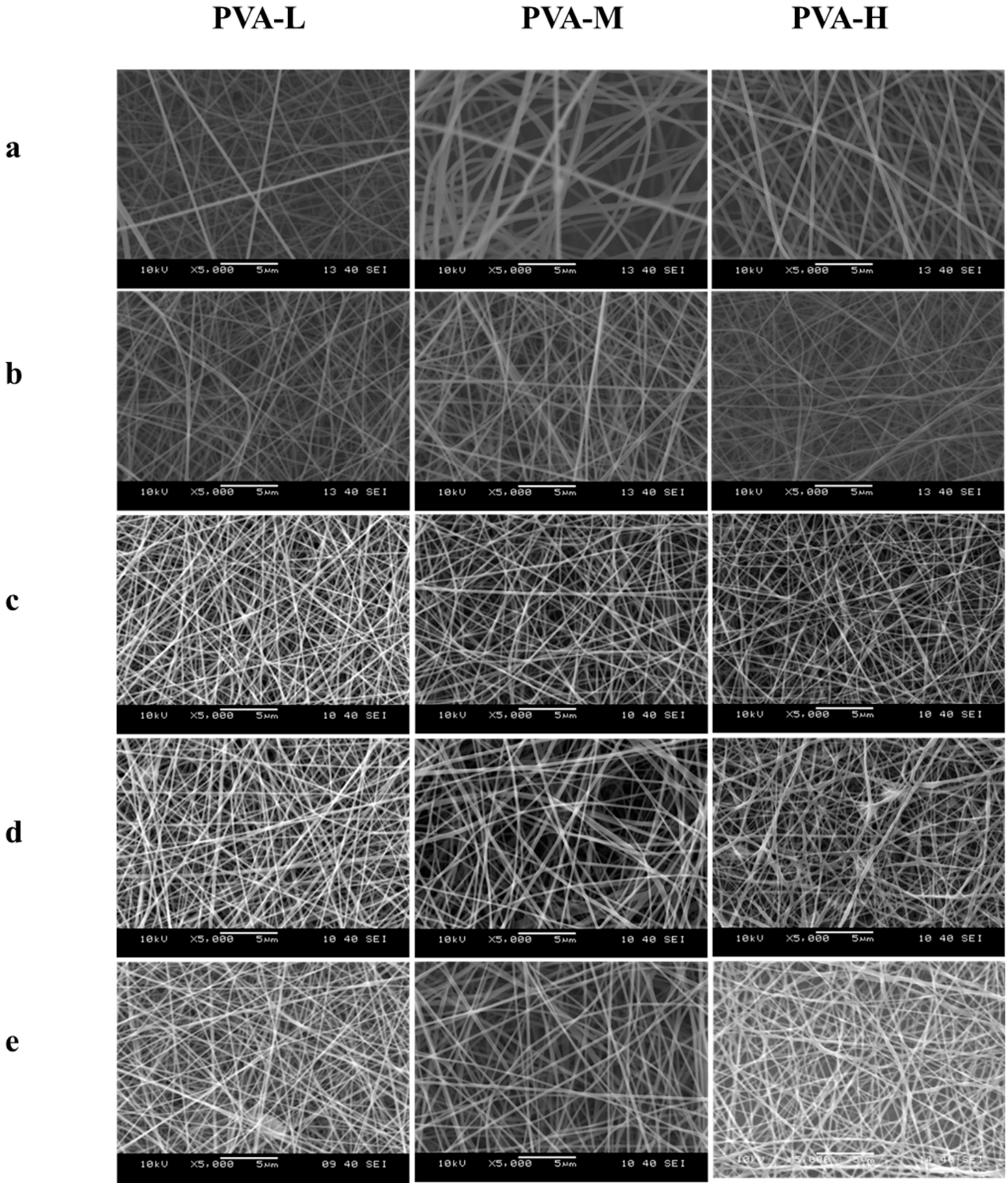

Figure S10. Scanning Electron Microscope (SEM) images of electrospun nanofibers obtained at 10 kV Magnification:5000x

PVA-L, PVA-M, and PVA-H; Polyvinyl alcohol (PVA) of Mw~67 kDa, Mw~130 kDa, and Mw~205 kDa, respectively). (a: plain PVA, b: polyvinyl alcohol and poloxamer 407 (PO-407) mixture 80:20), c: 1.1%(w/w) cysteamine hydrochloride (CysH) in PVA/PO-407 matrix, d: component of c plus EDTA 0.01 %(w/w), e: component of d plus polysorbate 80 (PS-80) 1%(w/w))

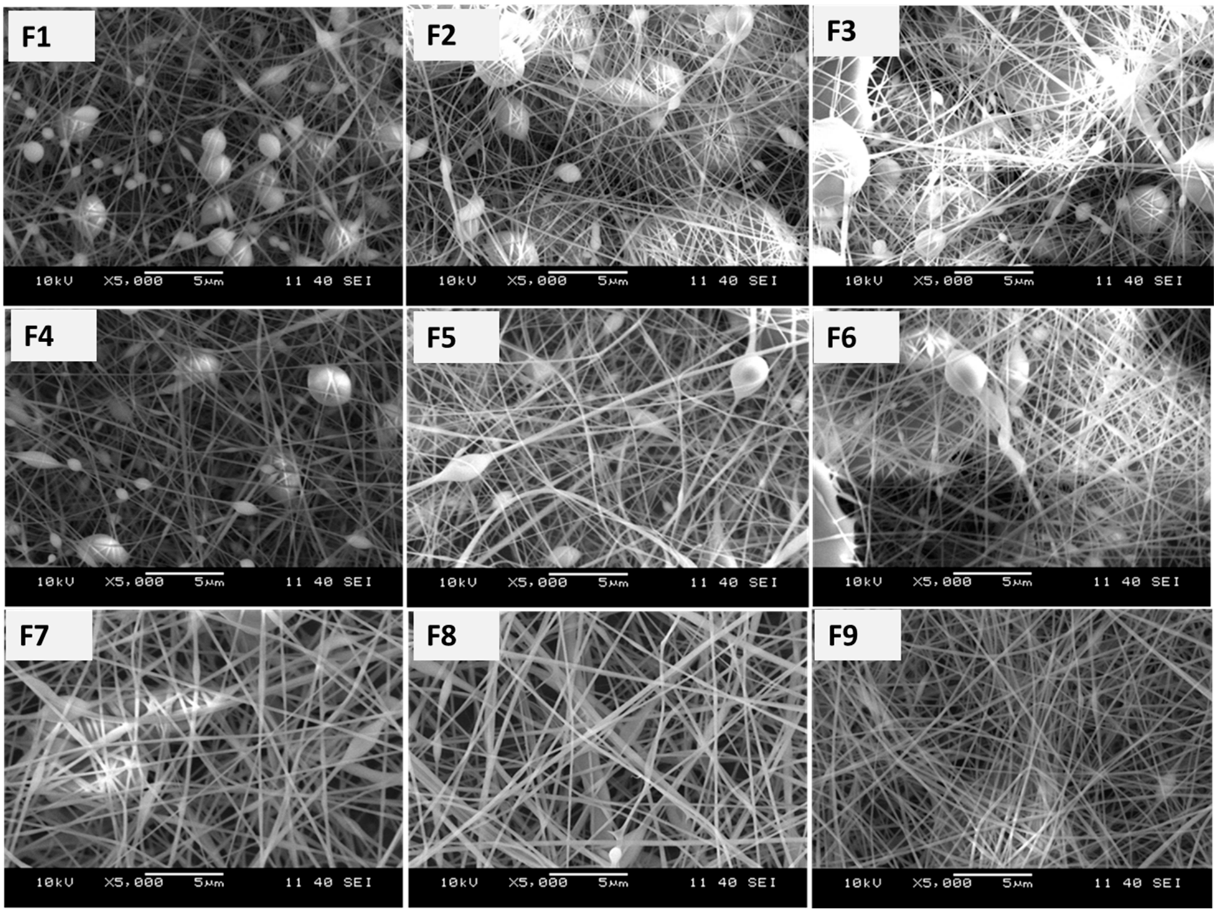

Figure S11. Scanning Electron Microscope (SEM) images of electrospun samples of different ratios of tetraethoxysilane (TEOS)/polyvinyl alcohol (PVA) mixture (magnification: 5000×)

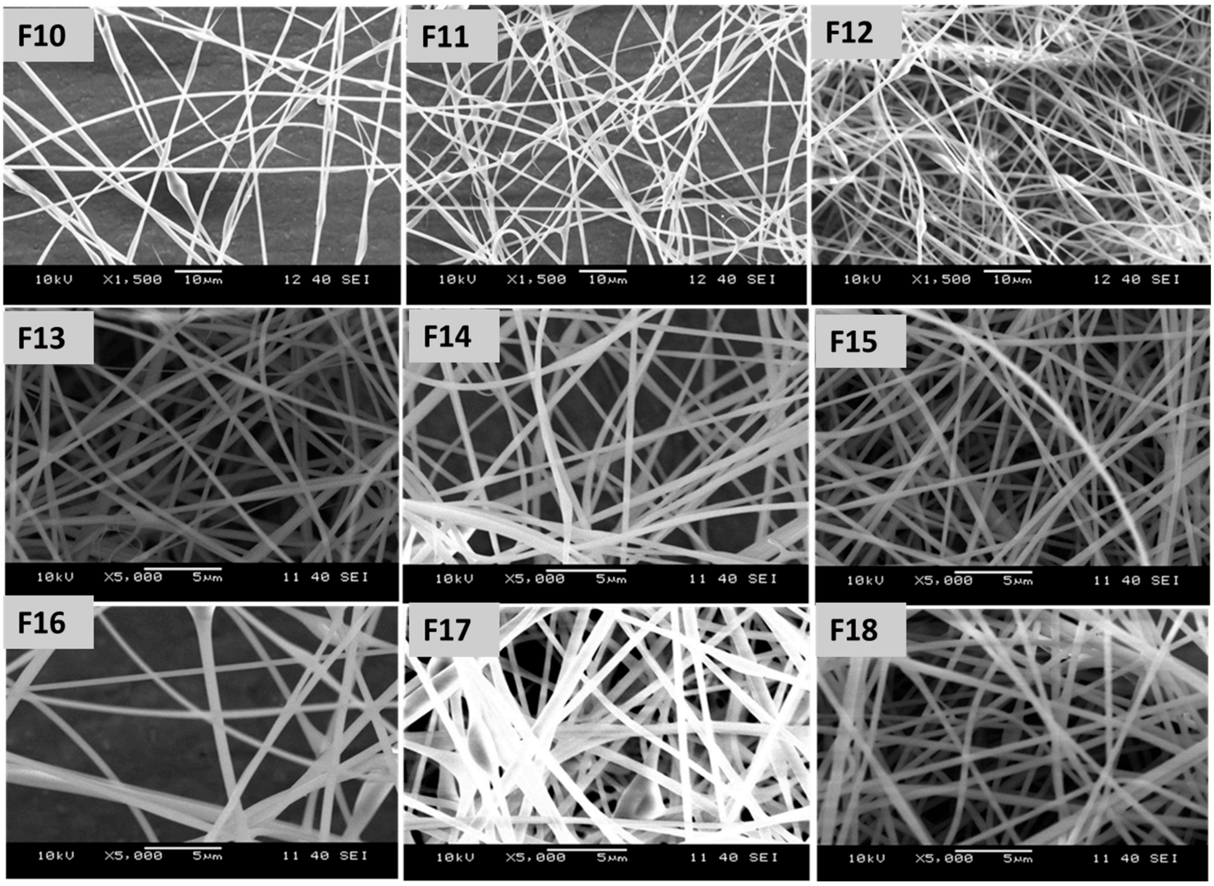

Figure S12. Scanning Electron Microscope (SEM) images of electrospun samples of different ratios of tetraethoxysilane (TEOS)/ polyvinyl alcohol (PVA) mixture (magnification: 5000×)

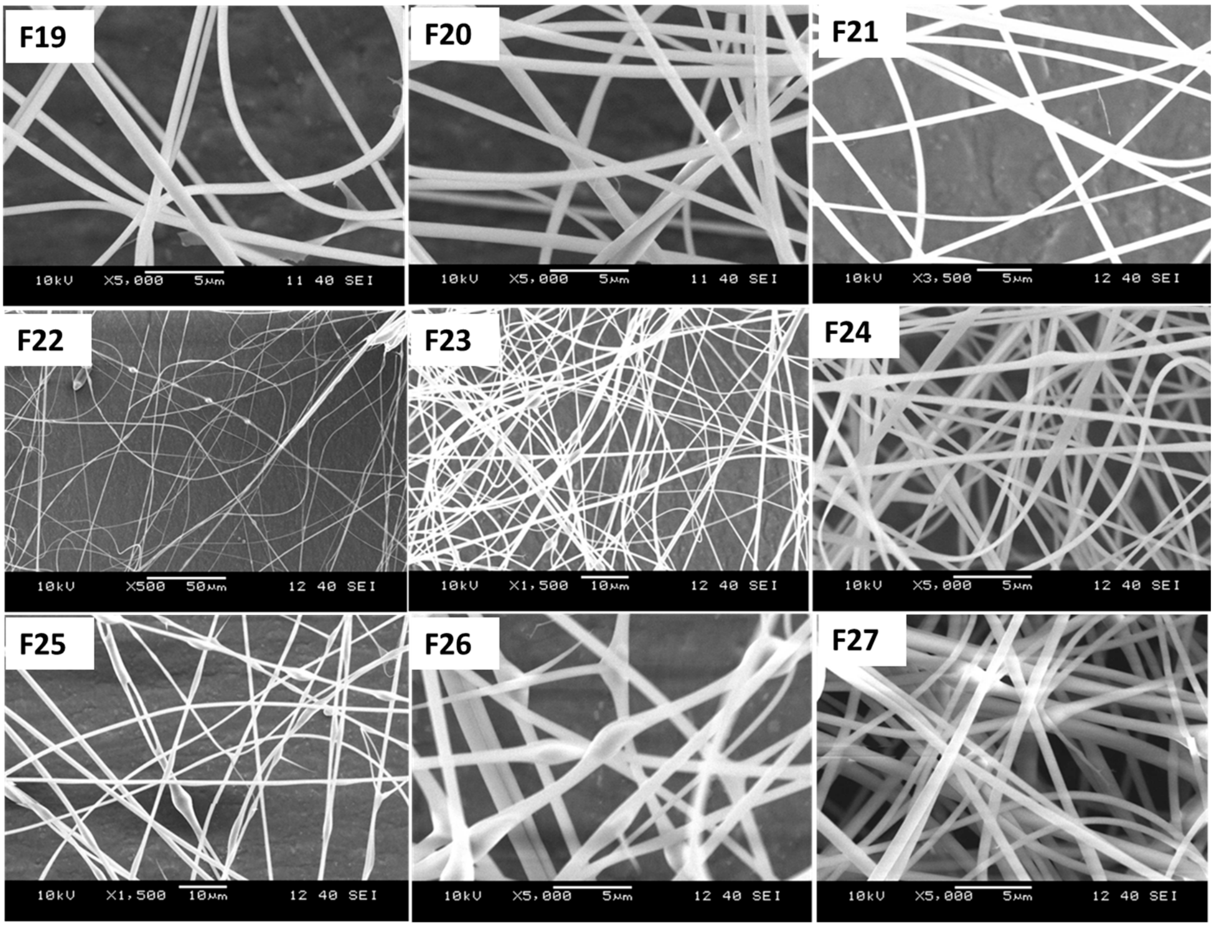

Figure S13. Scanning Electron Microscope (SEM) images of electrospun samples of different ratios of tetraethoxysilane (TEOS)/polyvinyl alcohol (PVA) mixture (magnification: 5000×)

Table S9. Average fiber diameters of polyvinyl alcohol (PVA) nanofibers with respective skewness and kurtosis

PVA grades	Formulation Code	Polymer concentration (% (w/w))	Average fiber diameter (nm) ± SD (nm)	Skewness	Kurtosis
Mw~67 kDa	P1	15	183 ± 27	0.648329	0.00709
	P2	17.5	190 ± 25	0.12191	0.055786
	P3	20	262 ± 34	-0.44344	-0.16322
Mw~130 kDa	P4	13	283 ± 39	0.396731	-0.35636
	P5	14	324 ± 36	0.29524	-0.23358
	P6	15	389 ± 47	0.093752	-0.52959
Mw~205 kDa	P7	5	170 ± 20	0.472034	-0.05075
	P8	7.5	296 ± 37	0.153405	-0.15343
	P9	10	425 ± 46	0.090873	-0.16408

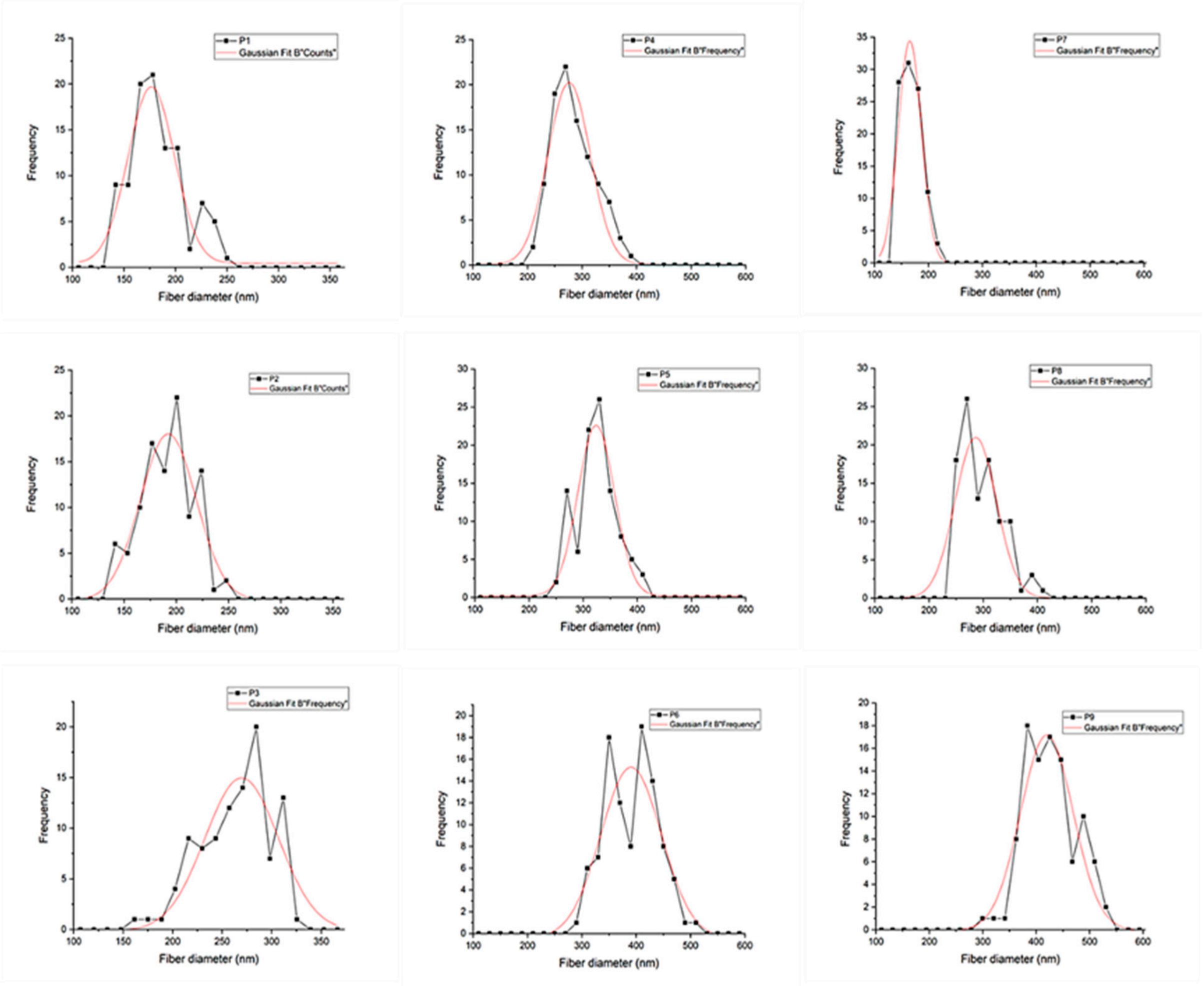

Figure S14. Diameter distribution curves of polyvinyl alcohol (PVA) nanofibers together with their Gaussian fitting. Samples were prepared from different grades of PVA. Where P1, P2 and P3 (Mw~67 kDa); P4, P5 and P6 (Mw~130 kDa); P7, P8 and P9 (Mw~205 kDa)

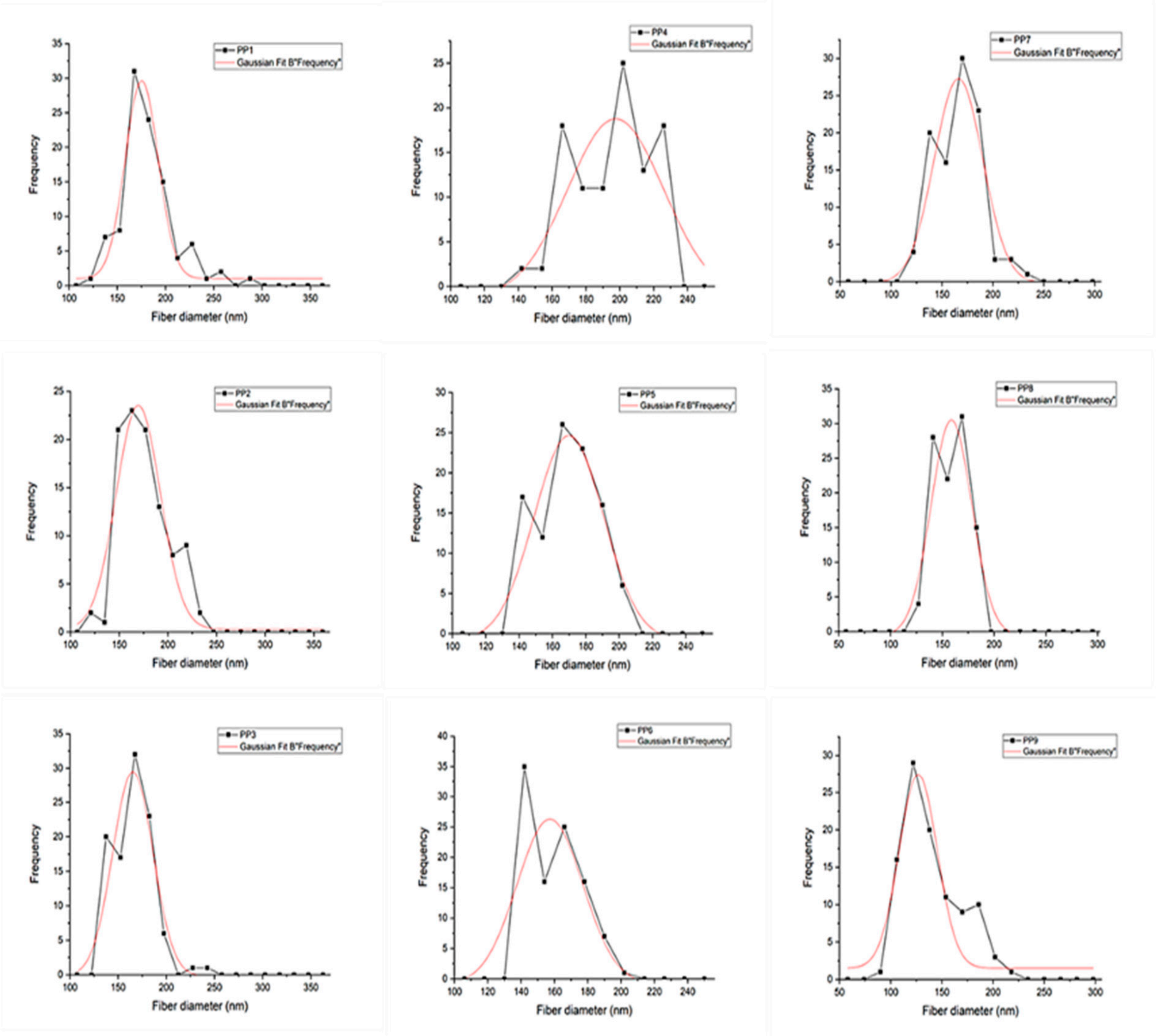

Figure S15. Diameter distribution curves of polyvinyl alcohol (PVA)/ poloxamer 407 (PO-407) nanofibers together with their Gaussian fitting. Samples were prepared from different grades of PVA. Where PP1, PP2 and PP3 (Mw~67 kDa); PP4, PP5 and PP6 (Mw~130 kDa); PP7, PP8 and PP9 (Mw~205 kDa) all PVA: poloxamer 407 (PO-407) ratios of 85:15, 80:20 and 75:25 respectively.

Table S10. Average fiber diameters of polyvinyl alcohol (PVA)/poloxamer 407 (PO-407) nanofibers with respective skewness and kurtosis

Formulation Code	PVA Grades	Total polymer Concentration (%w/w)	PVA: PO-407 (m:m)	Average fiber diameter (nm) ± SD (nm)	Skewness	Kurtosis
PP1	Mw~67 kDa	17.5	85:15	182 ± 28	1.204267	2.429205
PP2			80:20	173 ± 25	0.352606	-0.04599
PP3			75:25	167 ±19	0.847253	1.54499
PP4	Mw~130 kDa	14.0	85:15	195 ± 22	-0.28943	-0.78801
PP5			80:20	169 ±17	-0.17367	-0.9992
PP6			75:25	160 ± 16	0.325795	-1.2746
PP7	Mw~205 kDa	7.5	85:15	165 ±21	0.613269	1.345025
PP8			80:20	158 ± 15	0.216803	-0.87967
PP9			75:25	139 ± 29	0.418524	-0.48121

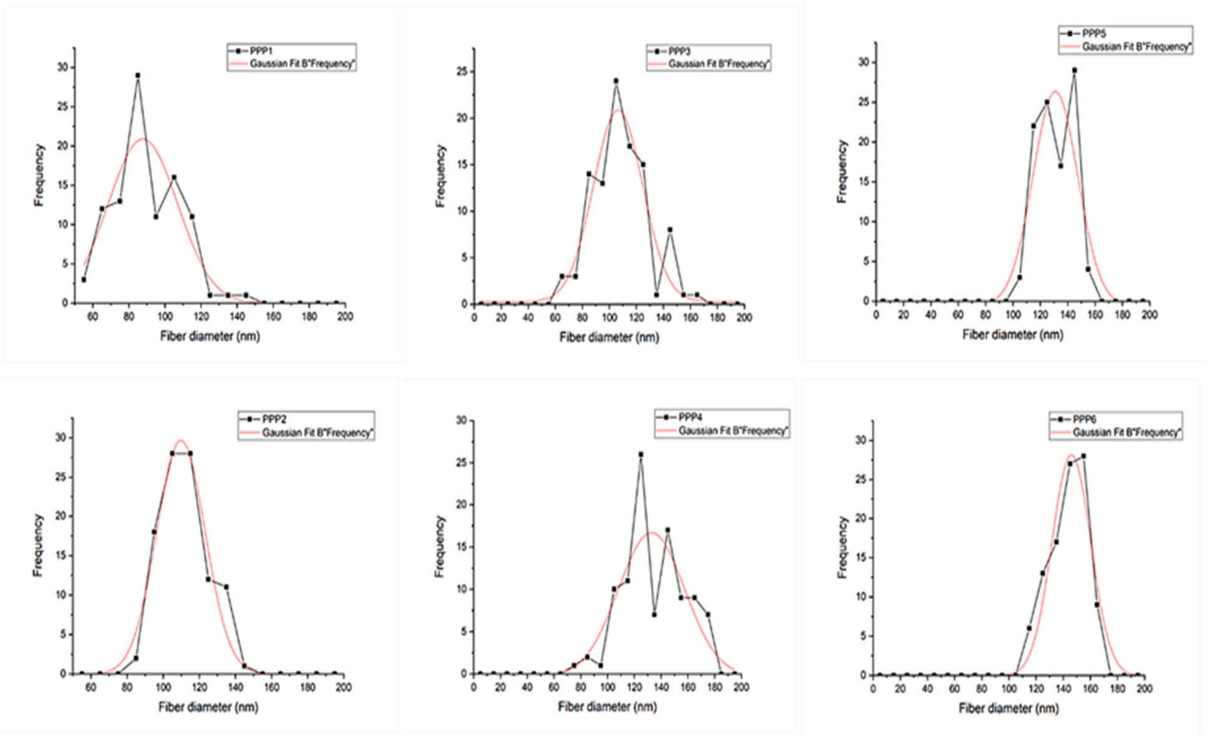

Figure S16. Diameter distribution curves of polyvinyl alcohol (PVA)/ poloxamer 407 (PO-407)/polysorbate 80 (PS-80) nanofibers together with their Gaussian fitting. Samples were prepared from different grades of PVA. Where PPP1 and PPP2 (Mw~67 kDa); PPP3 and PPP4 and PP6 (Mw~130 kDa); PPP5 and PPP6 (Mw~205 kDa). PVA: PO-407 ratio of 80:20 and PS-80 concentration of 1 (%(w/w)) and 0.5 (%(w/w)) respectively

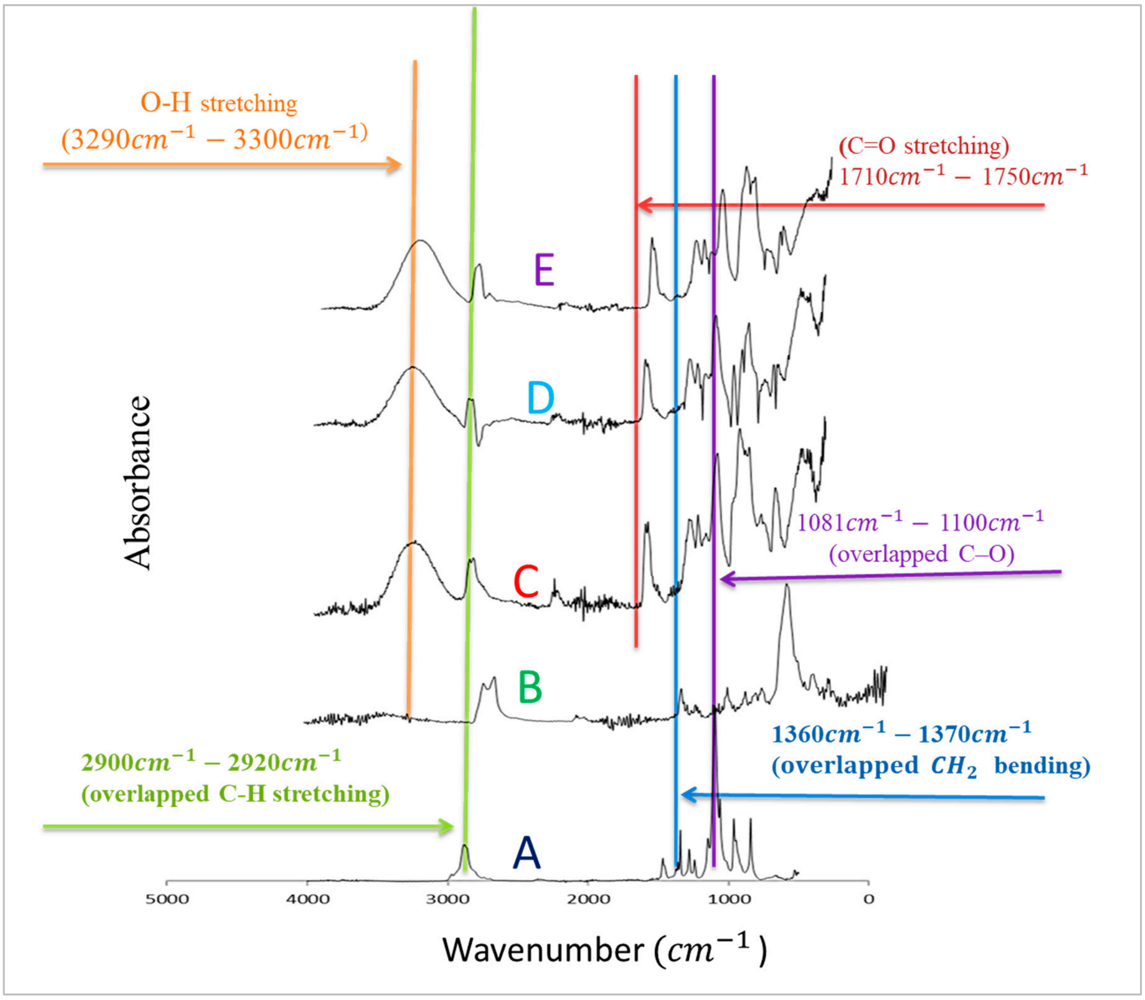

Figure S17. Fourier transform infrared (FTIR) spectra of (A) Poloxamer 407 (PO-407); (B) polysorbate 80 (PS-80); (C) polyvinyl alcohol (PVA) (powder); (D): PVA (Nanofibers) and (E): PVA/ PO-407/PS-80 blend. The PVA grade used was intermediate molecular weight (Mw~130 kDa)

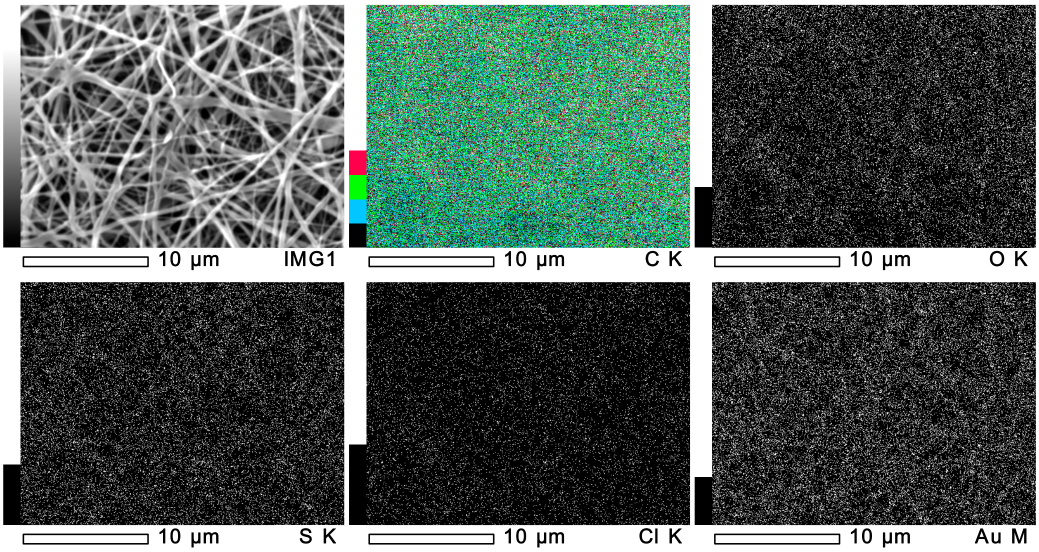

Figure S18. Energy dispersive X-ray spectroscopy (EDAX) analysis with mapping of F11 nanofibrous formulation (Cysteamine (Cys) 0.55 % (w/w) loaded in polyvinyl alcohol (PVA): poloxamer 407 (PO-407) mixture 80:20 mass ratio, with addition of ethylenediaminetetraacetic (EDTA) 0.01 %(w/w), and polysorbate 80 (PS-80) 0.5%(w/w)). Analysis was carried out at accelerated voltage of 15 KV. Despite the gold coating, these maps were valuable for identifying the location and relative abundance of carbon, oxygen, sulfur (thiol group of cysteamine), and chloride.

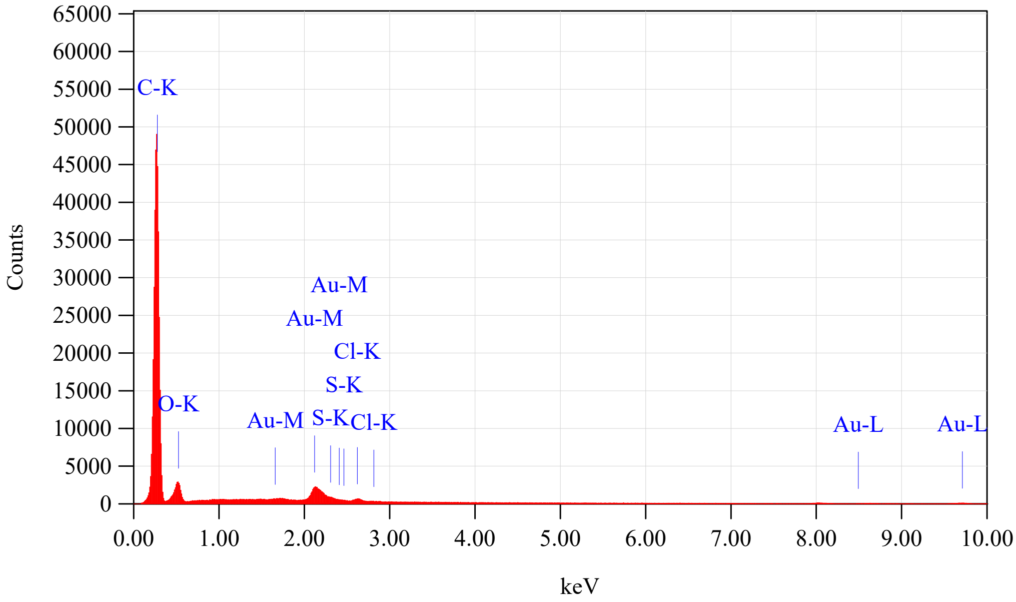

Figure S19. Energy dispersive X-ray spectroscopy (EDAX) spectra of F11 nanofibrous formulation (Cysteamine (Cys) 0.55% (w/w) loaded in polyvinyl alcohol (PVA): poloxamer 407 (PO-407) mixture 80:20 mass ratio, with addition of ethylenediaminetetraacetic (EDTA) 0.01 %(w/w), and polysorbate 80 (PS-80) 0.5%(w/w)). Analysis was carried out at accelerated voltage of 15 KV. Despite the gold coating, these maps were valuable for identifying the location and relative abundance of carbon, oxygen, sulfur (thiol group of cysteamine), and chloride.

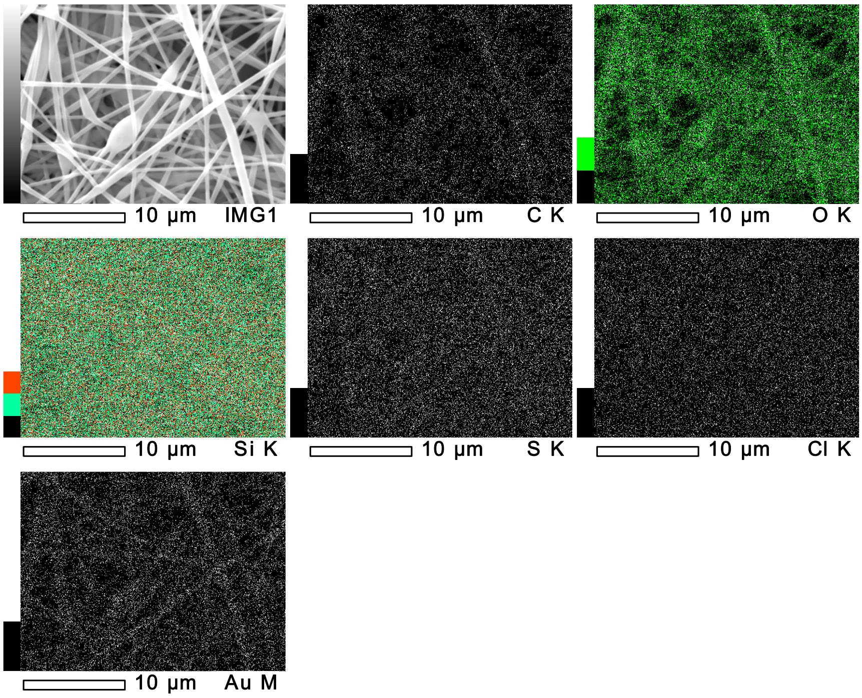

Figure S20. Energy dispersive X-ray spectroscopy (EDAX) analysis with mapping of FT27 nanofibrous formulation (Cysteamine (Cys) 1.1% (w/w) loaded in tetraethoxysilane (TEOS)/polyvinyl alcohol (PVA), TEOS: PVA of 4: 1 mass ratio). Analysis was carried out at accelerated voltage of 15 KV. Despite the gold coating, these maps were valuable for identifying the location and relative abundance of carbon, oxygen, sulfur (thiol group of cysteamine), silicon (silica), and chloride.

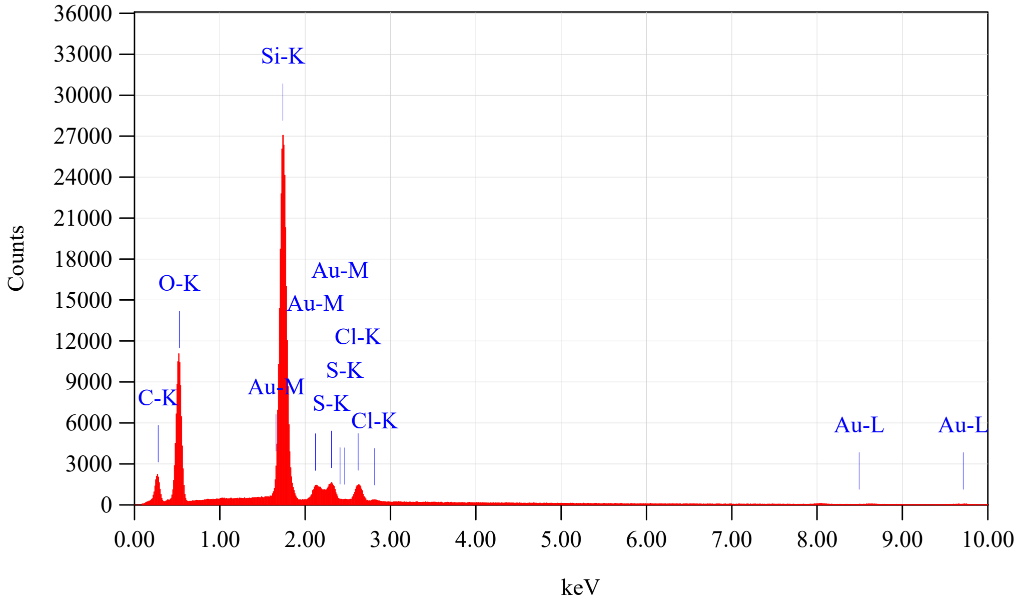

Figure S21. Energy dispersive X-ray spectroscopy (EDAX) spectra of FT27 nanofibrous formulation (Cysteamine (Cys) 1.1% (w/w) loaded in tetraethoxysilane (TEOS)/polyvinyl alcohol (PVA), TEOS: PVA of 4: 1 mass ratio). Analysis was carried out at accelerated voltage of 15 KV. Despite the gold coating, these maps were valuable for identifying the location and relative abundance of carbon, oxygen, sulfur (thiol group of cysteamine), silicon (silica), and chloride.

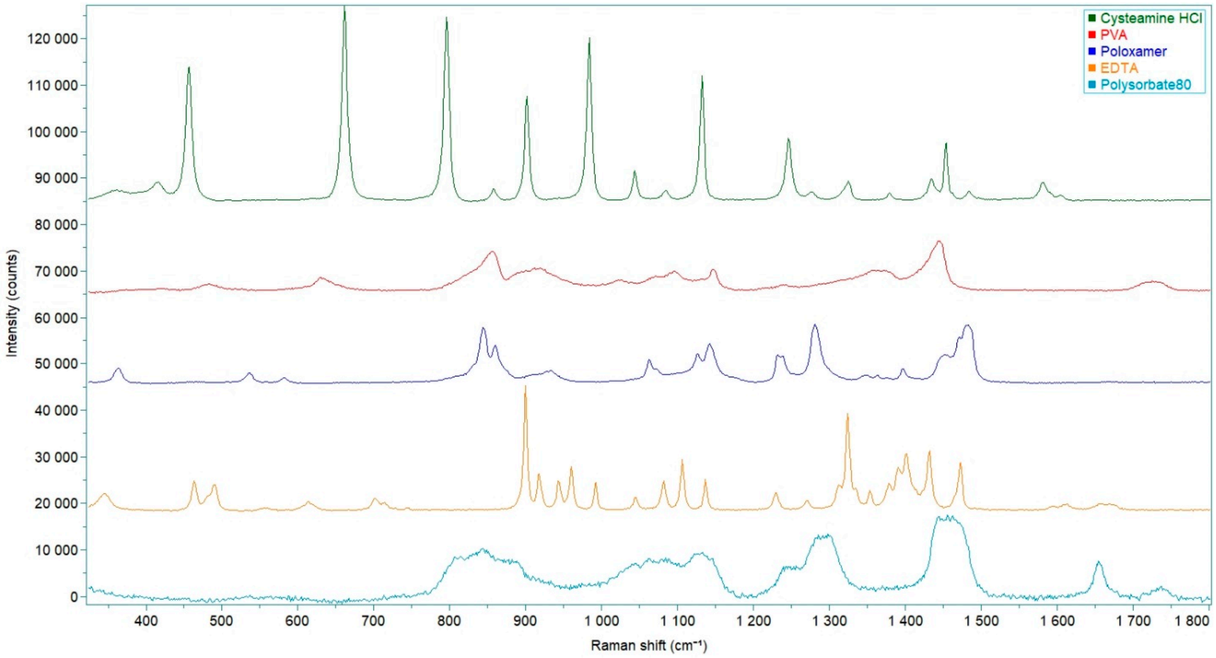

Figure S22. Raman spectra of the reference materials used for the fiber formation process (cysteamine HCl, polyvinyl alcohol (PVA), poloxamer 407, ethylenediaminetetraacetic (EDTA), and polysorbate 80).

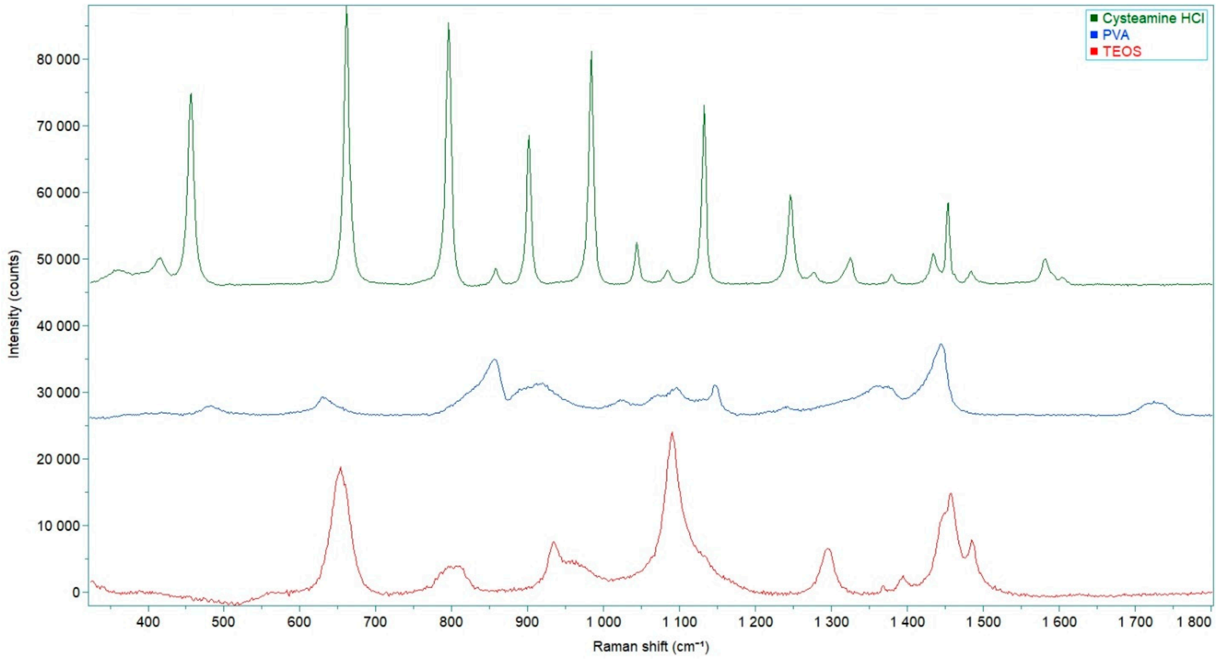

Figure S23. Raman spectra of the reference materials used for the fiber formation process (cysteamine HCl, polyvinyl alcohol (PVA), and tetraethoxysilane (TEOS)).

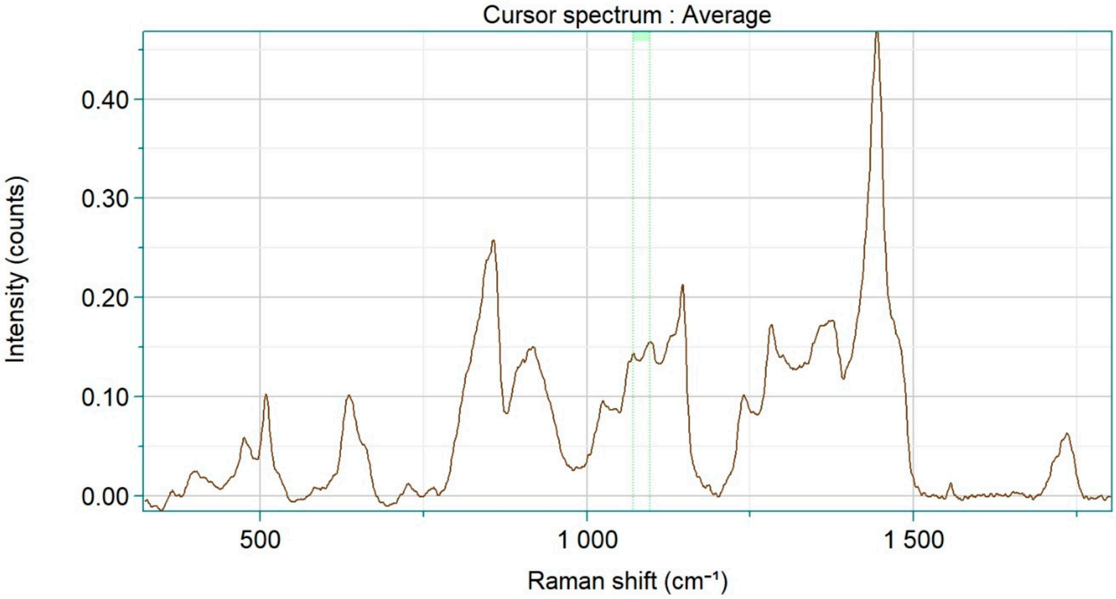

Figure S24. Raman spectra of F11 nanofibrous formulation (Cysteamine (Cys) 0.55% (w/w) loaded in polyvinyl alcohol (PVA): poloxamer 407 (PO-407) mixture 80:20 mass ratio, with addition of ethylenediaminetetraacetic (EDTA) 0.01 %(w/w), and polysorbate 80 (PS-80) 0.5%(w/w)).

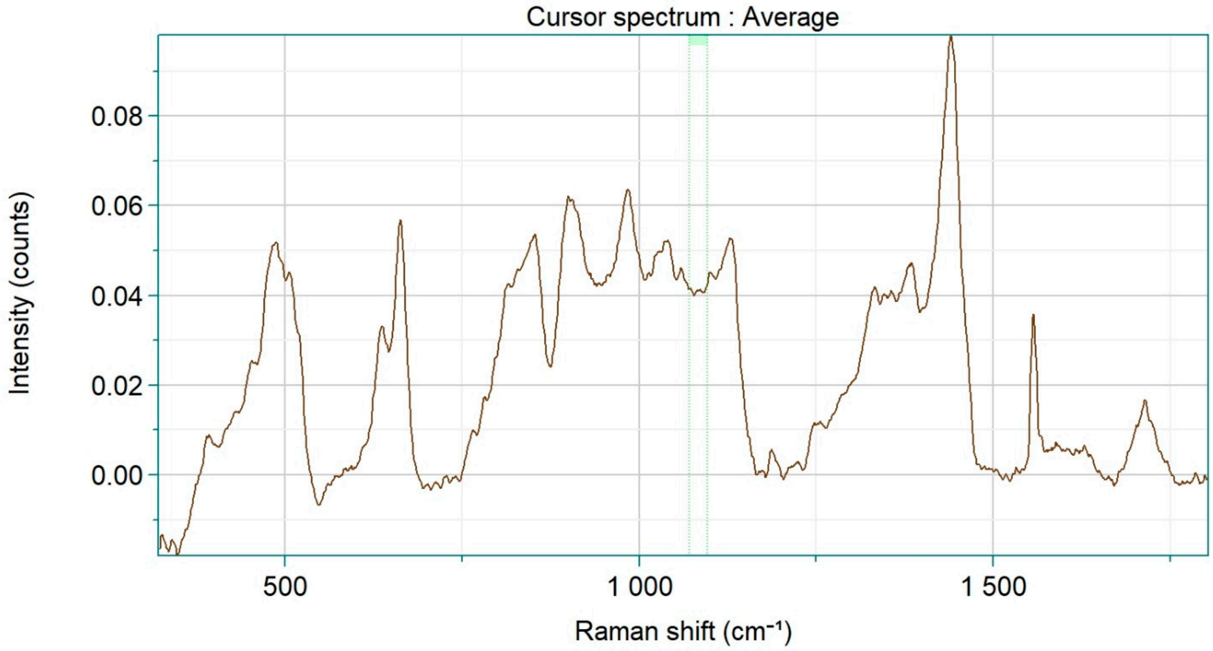

Figure S25. Raman spectra of FT27 nanofibrous formulation (Cysteamine (Cys) 1.1% (w/w) loaded in tetraethoxysilane (TEOS)/polyvinyl alcohol (PVA), TEOS: PVA of 4: 1 mass ratio)

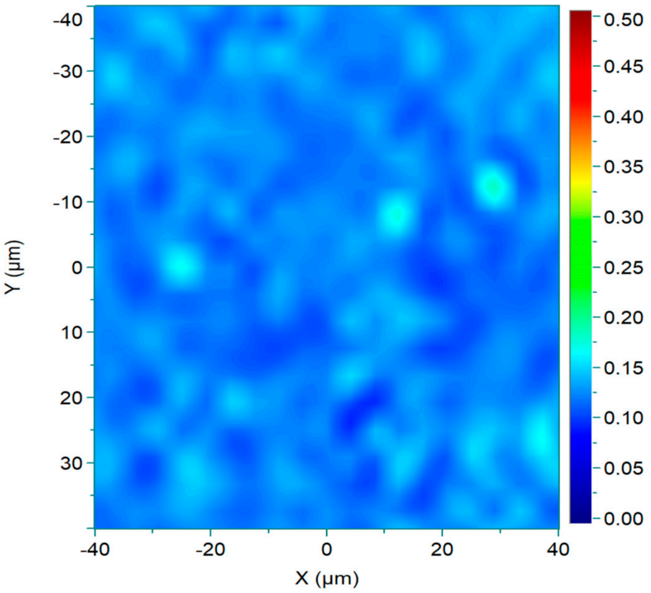

Figure S26. The Raman distribution of cysteamine in the polyvinyl alcohol/ poloxamer 407-based nanofibers. The high concentration of Cys is reflected by the red color, the green color reflects lower concentration.

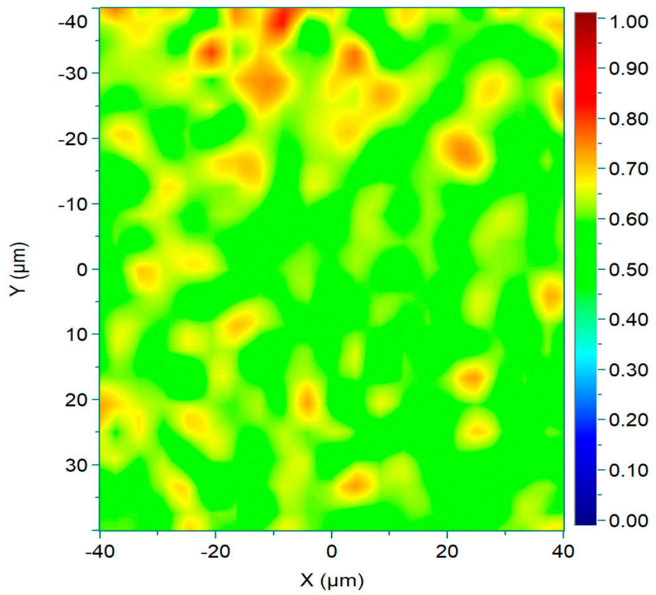

Figure S27. The Raman distribution of cysteamine in the tetraethoxysilane (TEOS)/polyvinyl alcohol (PVA)-based nanofibers. The high concentration of Cys is reflected by the red color, the green color reflects lower concentration.

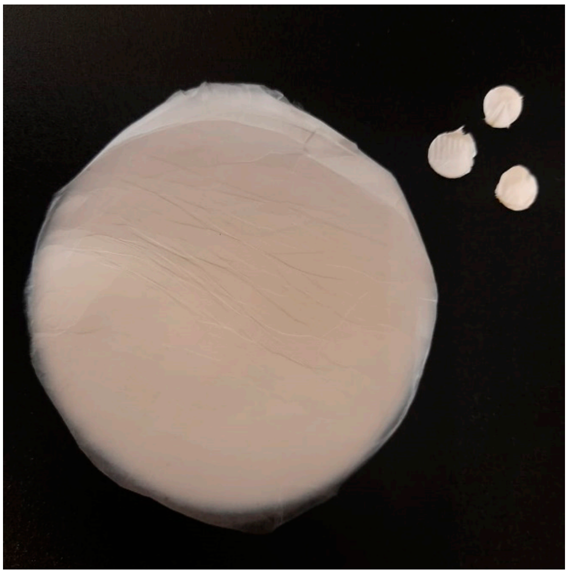

Figure S28. The images were taken from the electrospun nanofibrous samples
